# Mortality and re-fracture rates in low trauma hip fracture

**DOI:** 10.1186/s12877-024-04950-1

**Published:** 2024-04-30

**Authors:** Vahideh Mohseni, Noushin Fahimfar, Akram Ansarifar, Safdar Masoumi, Mahnaz Sanjari, Kazem Khalagi, Abolfazl Bagherifard, Bagher Larijani, Leila Janani, Mohammad Javad Mansourzadeh, Afshin Ostovar, Masoud Solaymani-Dodaran

**Affiliations:** 1 Department of Epidemiology, School of Public Health, Iran University of Medical Sciences, Tehran, Iran; 2 Osteoporosis Research Center, Endocrinology and Metabolism Clinical Sciences Research Institute, Tehran University of Medical Sciences, Tehran, Iran; 3 Department of Epidemiology and Biostatistics, School of Public Health, Tehran University of Medical Sciences, Tehran, Iran; 4 Department of Biostatistics, Faculty of Medical Sciences, Tarbiat Modares University, Tehran, Iran; 5 Obesity and Eating Habits Research Center, Endocrinology and Metabolism Clinical Sciences Institute, Tehran University of Medical Sciences, Tehran, Iran; 6 Bone and Joint Reconstruction Research Center, Shafa Orthopedic Hospital, Iran University of Medical Sciences, Tehran, Iran; 7 Endocrinology and Metabolism Research Center, Endocrinology and Metabolism Clinical Sciences Institute, Tehran University of Medical Sciences, Tehran, Iran; 8 Department of Biostatistics, School of Public Health, Iran University of Medical Sciences, Tehran, Iran; 9 Minimally Invasive Surgery Research Center, Hazrat-e-Rasool Hospital, Iran University of Medical Science, Tehran, Iran

**Keywords:** Osteoporosis, Hip fracture, One-year survival, Incidence, Death, Re-fracture

## Abstract

**Objectives:**

This study aimed to estimate the incidence rate of re-fracture and all-cause mortality rate in patients with hip fractures caused by minor trauma in the first year following the event.

**Materials and methods:**

This is a retrospective cohort study of patients over 50 years of age conducted in a referral hospital located in Tehran (Shafa-Yahyaian). Using the hospital information system (HIS), all patients hospitalized due to hip fractures caused by minor trauma during 2013–2019 were included in the study. We investigated the occurrence of death and re-fracture in all patients one year after the primary hip fracture.

**Results:**

A total of 945 patients with hip fractures during a 307,595 person-days of follow-up, were included. The mean age of the participants was 71 years (SD = 11.19), and 533 (59%) of them were women. One hundred forty-nine deaths were identified during the first year after hip fracture, resulting in a one-year mortality rate of 17.69% (95% CI: 15.06–20.77). The one-year mortality rate was 20.06% in men and 15.88% in women. Out of all the participants, 667 answered the phone call, of which 29 cases had experienced a re-fracture in the first year (incidence rate = 5.03%, 95% CI: 3.50–7.24). The incidence rates in women and men were 6.07% and 3.65%, respectively.

**Conclusion:**

Patients with low-trauma hip fractures have shown a high rate of mortality in the first year. Considering the increase in the incidence of hip fractures with age, comprehensive strategies are needed to prevent fractures caused by minor trauma in the elderly population.

**Supplementary Information:**

The online version contains supplementary material available at 10.1186/s12877-024-04950-1.

## Introduction

Hip fractures are common health issues and their frequency of occurrence is an indicator of the burden of osteoporosis. Although hip fractures account for less than 20% of all osteoporotic fractures, they are responsible for most of the fracture-related healthcare utilization and costs [1]. The occurrence of hip fractures is increasing in Asian, South American, and African countries. Although currently, the rate of hip fractures in these countries is lower than the rate of European and North American countries, it will surpass them by 2050 [2]. Approximately 30% of hip fractures occur in men, and mortality, morbidity, and loss of independence after hip fractures are more significant in men than in women [3, 4].

Osteoporosis, characterized by low bone mass with microarchitectural deterioration of bone tissue, intensifies with age, and is associated with bone fragility [3, 5]. Fractures in the elderly are usually caused by a combination of falls and osteoporosis [6]. Fractures that occur due to minor trauma can be a sign of osteoporosis. A person with osteoporosis is at high risk of re-fractures in the first two years after the initial fractures [7]; and the risk of death is also higher for these people. Osteoporotic fractures decrease the quality of life and increase the rate of mortality with a significant financial burden on health systems [8].

Osteoporosis treatment can reduce the risk of re-fracture by 50% [9, 10]. Therefore, it is necessary to identify people at risk of re-fracture and start osteoporosis treatment immediately [9, 10]. The effectiveness of osteoporosis treatment in preventing the occurrence of secondary fractures could be diminished by irregular use of anti-osteoporosis drugs [11]. Unfortunately, adherence to osteoporosis treatment is poor and most patients stop treatment within the first year [12]. Therefore, long-term treatment management with a personalized approach is needed to treat osteoporosis [13].

Failure to act to prevent re-fracture is a pervasive problem. Despite improvements in the diagnosis and treatment methods of osteoporosis, there has not been a significant reduction in the occurrence of secondary fractures [1, 14]. The high prevalence of osteoporosis in women has made hip fracture more of a health concern in women, requiring gender-specific approaches to its epidemiology and management. In this retrospective cohort study of people over 50 who suffered low-trauma hip fractures, we estimated the incidence rate of re-fracture and all-cause mortality by gender during the first year in Iran.

## Materials and methods

This is a retrospective cohort study of patients over 50 years of age conducted in a referral hospital (Shafa-Yahyaian) in Tehran. All patients hospitalized due to low-trauma hip fractures during 2013–2019 were included in the study. We defined low-trauma hip fracture as one resulting from a fall while standing up or walking, falling from standing height or less, or falling from stairs or slipping. The status of life or death and the occurrence of re-fracture during one year after the initial hip fracture were investigated.

To collect information, a questionnaire was prepared that included two parts. The first part, including demographic information and information related to the patient’s hospitalization, was completed using the patient’s electronic or paper file in the hospital. The second part was set up for use during a telephone interview, in which questions were asked about life status, re-fracture, and treatment of osteoporosis. To complete the first part of the questionnaire, the electronic records of patients with hip fractures that met the operational definition of ICD classification codes, including S720 femoral neck fracture, S721 intertrochanteric fracture, S722 sub-trochanteric fracture, and non-traumatic fracture, were obtained from the health information system (HIS) of the hospital and the contents of their hospital records were reviewed. The patient-related data retrieved from medical records, including demographic information, underlying diseases, location of the fracture, type of trauma, type of treatment, and duration of hospitalization, were extracted and recorded. In case of missing information in the HIS system of the hospital, the required information was completed using the patients’ paper files.

To complete the second part of the questionnaire, telephone interviews were conducted with all patients. If the patient was unable to do a telephone interview, the questions were asked from a close relative or the nurse responsible for the patients’ care. If the patient missed a phone call, the nurse repeated the call up to three times. First, the patients or interviewees were asked about the status of life or death and the occurrence of re-fracture. Then, if the respondent was the patient or someone informant about the patient’s situation, additional information was asked, including the patient’s awareness of osteoporosis and ways to diagnose and treat it, as well as whether he or she had the disease. The death records was also retrieved from the national death registry database in the Ministry of Health using the unique national code.

We described the basic characteristics of the participants by gender, using mean, median, and standard deviation for quantitative, and frequencies for qualitative variables. The cumulative incidence rate of re-fracture and death were obtained by dividing the number of deaths or re-fractures during the study by the person-time of the population at risk. We calculated 95% confidence intervals for the rates based on poisson distribution. The survival rate and fracture-free survival (when the event is re-fracture) in men and women were evaluated through the survival table, and the Kaplan-Meier curve. Cox proportional hazard regression models were built to calculate hazard ratios and their 95% confidence intervals for potential risk factors. These factors were age, gender, fracture treatment approach, and comorbidities including history of hypertension, stroke, chronic kidney failure, thyroid disease, cancer, diabetes, and myocardial infarction. We took them into account in our regression models as they could independently increase the risk of mortality or re-fracture outcomes. Statistical analyses were performed using STATA version 14 software, and P-values ≤ 0.05 were considered statistically significant.

## Results

**Fig. 1:**
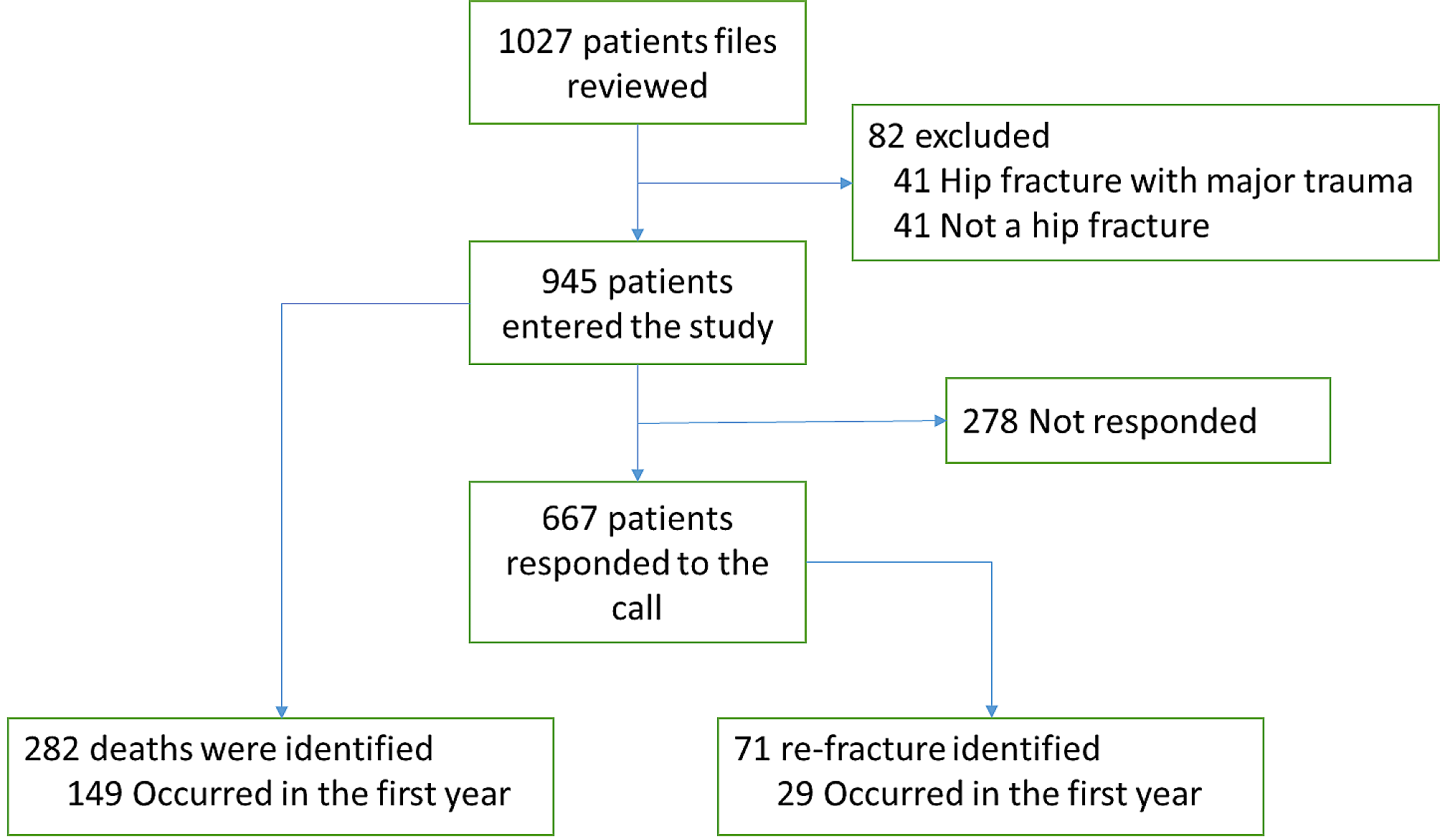
Flow diagram of participants in the retrospective cohort

A total of 1027 cases of patients over 50 years of age who suffered low-trauma hip fractures admitted to Shafa-Yahyaian Hospital between 2013 and 2019 were reviewed and 945 (533 women) eligible patients were recruited in the study (Fig. 1). The average age of the participants was 71 years (SD = 11.19). In this retrospective cohort study, the total follow-up was 1,070,717 person-days, and the median follow-up period was 1013 days. In these people, the most common places of fracture were the intertrochanteric fracture region with 477 cases (50.48%), and the neck femur with 428 cases (45.29%). In all, 588 patients (62.22%) were treated surgically. Of the patients who met the inclusion criteria, 30 cases (3.17%) had a history of receiving osteoporosis treatments, 163 cases (17.25%) were using calcium supplements and 166 cases (17.57%) were receiving vitamin D supplements. The summary of the participants’ characteristics is given in Table 1; Fig. 1.

**Table 1:** General characteristics of the cohort of patients with hip fractures caused by low trauma who were referred to Shafa-Yahyaian Hospital between 2013–2019 by gender

		Female *N* = 533	Male *N* = 412	Total *N* = 945
Age group				
	50–59	70 (13.13%)	88 (21.36%)	158 (16.72%)
	60–69	114 (21.39%)	111 (26.94%)	225 (23.81%)
	70–79	186 (34.90%)	100 (24.27%)	286 (30.26%)
	>=80	163 (30.58%)	113 (27.43%)	276 (29.21%)
Education				
	No education	241 (45.22%)	131 (31.80%)	372 (39.37%)
	Primary school	68 (12.76%)	54 (13.11%)	122 (12.91%)
	Secondary school	29 (5.44%)	50 (12.14%)	79 (8.36%)
	Diploma	25 (4.69%)	39 (9.47%)	64 (6.77%)
	College	14 (2.63%)	18 (4.37%)	32 (3.39%)
	Missing	156 (29.27%)	120 (29.13%)	276 (29.21%)
Fracture type				
	femoral neck	247 (46.34%)	181 (43.93%)	428 (45.29%)
	Intertrochanteric	264 (49.53%)	213 (51.70%)	477 (50.48%)
	Subtrochanteric	22 (4.13%)	18 (4.37%)	40 (4.23%)
Osteoporosis treatment				
	Received	23 (4.32%)	7 (1.70%)	30 (3.17%)
	Not received	510 (95.68%)	405 (98.30%)	915 (96.83%)
Vitamin D supplementation				
	Received	109 (20.45%)	57 (13.83%)	166 (17.57%)
	Not received	424 (79.55%)	355 (86.17%)	779 (82.43%)
Calcium supplementation				
	Received	106 (19.89%)	57 (13.83%)	163 (17.25%)
	Not received	427 (80.11%)	355 (86.17%)	782 (82.75%)
Fracture treatment approach				
	Surgical	323 (60.60%)	265 (64.32%)	588 (62.22%)
	Medical	4(0.75%)	10(2.43%)	14(1.48%)
	No information	206(38.65%)	137(33.25%)	343(36.30%)
Underlying diseases				
	Hypertension	160 (30.02%)	68 (16.50%)	228 (24.13%)
	Stroke	20 (3.75%)	21 (5.10%)	41 (4.34%)
	Myocardial infarction	3 (0.56%)	6 (1.46%)	9 (0.95%)
	Kidney disease	5 (0.94%)	4 (0.97%)	9 (0.95%)
	Thyroid disease	27 (5.07%)	12 (2.91%)	39 (4.13%)
	Cancer	18 (3.38%)	19 (4.61%)	37 (3.92%)
	Diabetes	84 (15.76%)	50 (12.14%)	134 (14.18%)
Death during the first year				
	Alive	457(85.74%)	339(82.28%)	796(84.23%)
	Dead	76(14.26)	73(17.72%)	149(15.77%)
Re-fracture during the first year				
	Re-fracture	20(5.33%)	9(3.08%)	29(4.35%)
	No Re-fracture	355(94.67%)	283(96.92%)	638(95.65%)

**Fig. 2:**
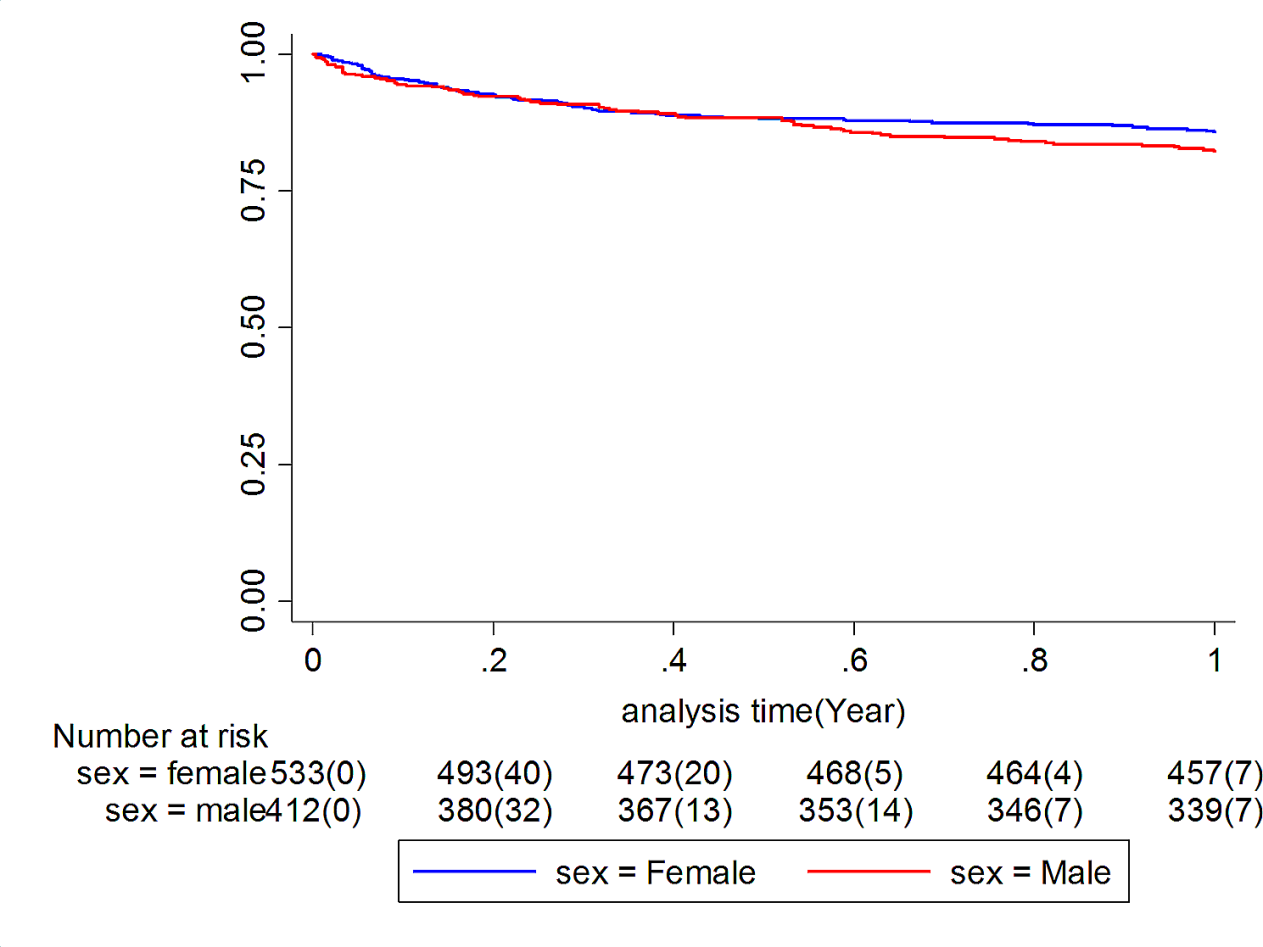
Kaplan-Meier survival curve for death by sex in the study cohort (Log Rank test: P-value < 0/001)

During this study, 282 deaths were observed (Fig. 1), of which 149 were in the first year. In this way, the one-year mortality rate is 17.69% (95% CI: 15.06–20.77). The mortality rate in men was 20.06% (95% CI: 9.15–24.25) which was higher than in women (15.88%, 95% CI: 12.68–19.89) (Table 2). Figure 2 shows the Kaplan-Meier survival curve in both male and female groups. Of the total deaths observed in the first year, five cases had a previous history of hip fracture. We showed that age, male sex, cancer, and diabetes are independent risk factors for death in the first year (Table S1 and S13).

**Table 2:** Mortality and re-fracture rates (percent) in the first year in the cohort of study participants by gender

		Person-day of follow up	Event	Rate per 100 per year	95% Confidence Interval
Death at the first year					
	Female	174,724	76	15.88	12.68–19.89
	Male	132,870	73	20.06	15.95–25.24
	Total	307,595	149	17.69	15.06–20.77
Re-fracture at the first year					
	Female	120,151	20	6.07	3.91–9.41
	Male	89,947	9	3.65	1.90–7.01
	Total	210,098	29	5.03	3.50–7.24

**Fig. 3:**
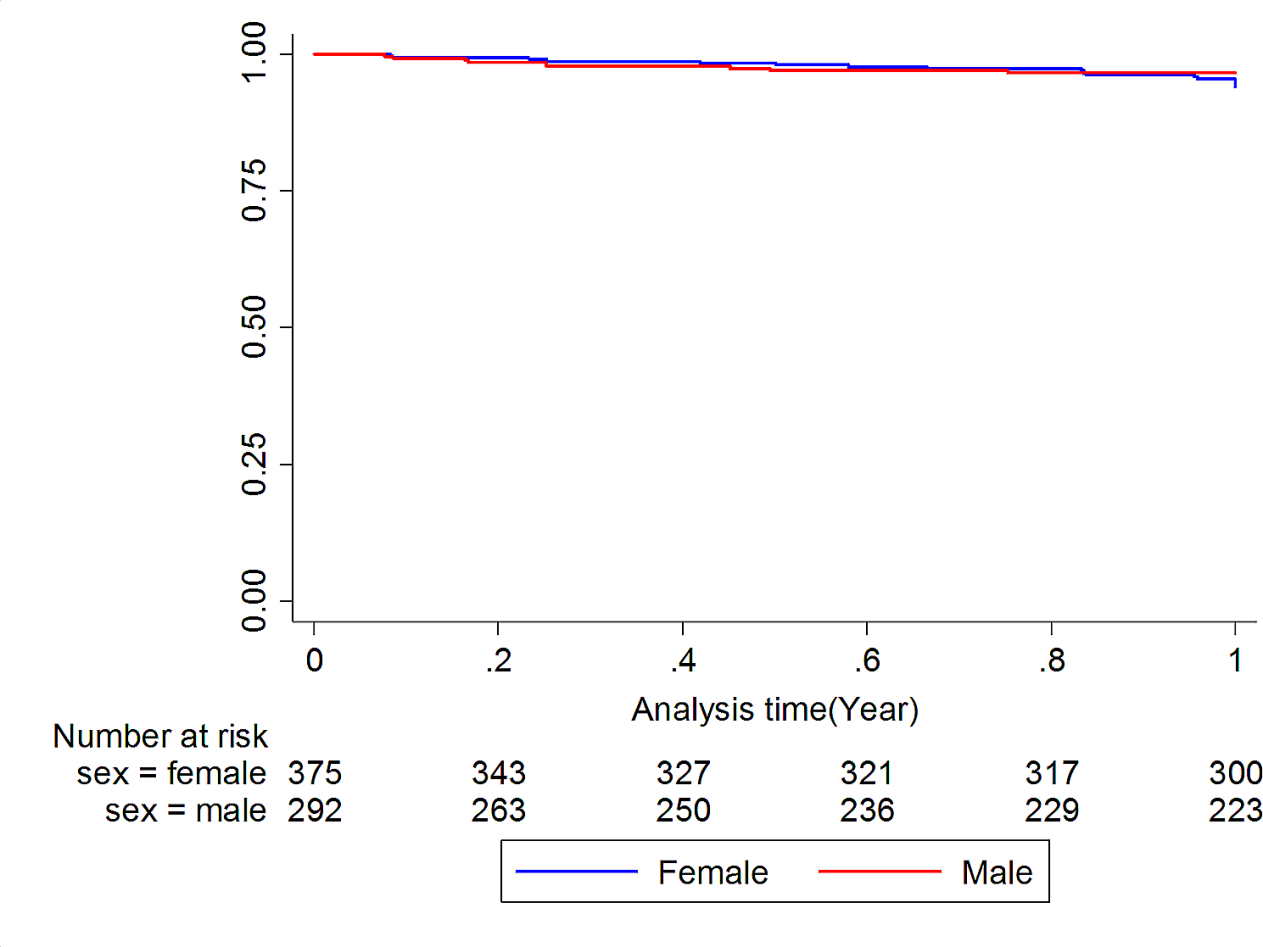
Kaplan-Meier curve of fracture-free survival for re-fracture by sex in the study cohort (Log Rank test: P-value = 0.16)

Of the 667 patients who answered the phone call, 71 cases had re-fractures (4.05%), of which 29 fractures (20 in women and 9 in men) occurred in the first year after the first fracture (Fig. 1). In this way, the re-fracture rate in the first year was calculated to be 5.03% (95% CI: 3.50–7.24). This rate was 6.07% in women (95% CI: 3.91–9.41) and 3.65% in men (95% CI: 1.90–7.01) (Table 2). Figure 3 shows the Kaplan-Meier curve of fracture-free survival for re-fracture in both male and female groups. We showed that cancer is an independent risk factor for re-fracture in the first year (Table S2 and S4).

## Discussion

Our study showed that the rate of re-fracture and mortality in the first year in a group of patients with low-trauma hip fractures referring to a specialized hospital was 5.03% and 17.69% respectively. Although the re-fracture rate in the first year was higher in women than in men, the mortality rate was lower.

Estimates of first-year mortality rate following hip fracture in different reports vary depending on the reporting period, country, and patient age. The 17.69% one-year mortality rate in our study is closer to the published reports from Asian countries [15–18] (9.2–24.2%) rather than the other parts of the world [19–33] (12.8–35.3%). (see Table 3). Over the past few decades, the mortality rates have shown a decreasing trend. In a review study that examined death data from 1981 to 2012 [32], this figure has decreased from 34% at the beginning of the period to 24%at the end of it. This decrease in mortality has likely been achieved by improving health conditions, providing more services for the elderly, and reducing the prevalence of hip re-fractures by increasing the awareness of patients about osteoporosis and its relationship with fracture. However, the mortality rate is still high, and it is necessary to plan for its further decrease.

**Table 3:** Summary of available reports of mortality and re-fracture within one year after hip fracture caused by minor trauma

No	Year	Region	Period	Population	Mortality 1st year (95% CI)	Re-fracture 1st year (95% CI)
1	1989	America (20)	1984–1986	814; >60	17.4	-
2	1993	melton (34)	1970–1985	3898;> 40	-	6.2
3	2000	Canada (21)	1995–1996	504; > 50	25.2	5.2
4	2000	Canada (21)	1995–1996	399; >50	25.2	5.2
5	2001	Thailand (18)	1997–1998	384; >50	24.2	-
6	2005	England (22)	1999–2003	2660; >50	30	-
7	2006	Italy (23)	200–2001	252; > 70	24	-
8	2007	Australia (37)	1985–2005	337; males > 60	-	3.47 (2.68–4.48)
9	2007	Australia (37)	1985–2005	905; females > 60	-	1.95 (1.70–2.25)
10	2007	Finland (47)	2002–2003	34; >60	-	5.08 (3.3–7.78)
11	2010	Thailand (17)	1998–2003	632; >50	18	-
12	2012	Canada (24)	2004–2008	761; males > 65	33	-
13	2012	Canada (24)	2004–2008	2241; females > 65	22	-
14	2012	Spain (25)	2005–2006	139; > 65	29.3	-
15	2013	S. Korea (48)	2003–2011	71; >50		2.4
16	2014	Norway (49)	1999–2008	7836; males > 50	4.6 (4.5–4.7)	-
17	2014	Norway (49)	1999–2008	12,153; females > 50	2.8 (2.8–2.9)	-
18	2014	Canada (32)*	1981–2012	13,379; >60	Early period 34; Late p. 24	-
19	2015	Austria (36)	2008–2010	2166; >50	-	2.97 (2.75–3.19)
20	2015	Thailand (15)	2013–2015	112; >50	9.2	-
21	2015	Sweden (26)	2006–2012	116,111; >50	25.9	-
22	2018	China (16)	2018	1050; >50	14.9	-
23	2019	Italy (27)	2015–2016	667; > 65	18.17	-
24	2019	Italy (28)	2013–2015	728; >65	16.6	-
25	2019	Austria (50)	2012–2016	2280; >50	35.3	4.8
26	2019	Canada (51)	2007–2010	6543	-	1.96
27	2019	Poland (33)	2008–2015	83,543; males > 50	ranged 30.45 to 32.8	-
28	2019	Poland (33)	2008–2015	205,687; females > 50	ranged 26.2 to 28	-
29	2019	Italy (28)	2013–2015	728; > 65	16.6	-
30	2020	Thailand (52)	2014–2018	1412; >50	19	-
31	2020	Italy (35)	2016–2017	289; > 65	-	14.2
32	2021	Europe (29)	2012–2016	888; > 50 years	21.2	-
33	2021	France (30)	2009–2014	55,831; >50	12.8 (12.7–12.9)	6.3 (6.2–6.3)
34	2021	Canada (31)	2011–2017	73; >65	14.4	-
35	2021	Sweden (53)	2018–2019	94; > 57	0.9 (0.4–2.2)	1 (0.3–3.5)

Studies have shown that, in the elderly, the risk of recurrent hip fractures during the first year after the initial event rises considerably associated with higher clinical vulnerability and mortality. In the current study, the incidence rate of re-fracture in the first year is estimated at 5.03% (6.07% in women and 3.65% in men). In a study conducted in Canada on 527 people aged 50 and over, the incidence rate of hip re-fracture was also at about 5.2% [21]. While this rate was reported as 1.7% in a study conducted in Denmark on a population of 3898 people aged 40 and over [34].In a study conducted in Italy in 2020, the incidence rate of re-fracture of the hip was reported as 14.6% and the insufficient coverage of osteoporosis treatments among these people was identified as the cause. Only 16.7% of these people had been treated with anti-osteoporosis drugs. In addition, it seems that the loss of independence and mobility after the first fracture plays an important role in increasing the rate of re-fracture [35]. Differences observed in the rate of re-fracture are related to the age distribution of people who participated in different studies, and for a more accurate comparison, age-standardized rates should be compared.

The results of our study showed that the relationship between first-year mortality and re-fracture rates in men and women differ; i.e. while the re-fracture rate is higher in women than in men, mortality is lower. This finding can be a reflection of the overall high mortality rate and lower life expectancy in men compared to women. Studies have shown that treatment of osteoporosis, whether started before or after hip fracture, reduces mortality from hip fracture [36]. In our study, women received more anti-osteoporosis drugs and vitamin D and calcium supplements than men (see Table 1), suggesting that men pay less attention to their health and this may have also contributed to their higher mortality.

We found that the re-fracture incidence rate was higher in women than in men. This finding is consistent with most of the available reports [37, 38]. The density of bone mass decreases more in women than in men beyond the reproductive age because of menopause which increases the frequency of falls and re-fractures. It seems that the decrease in the density of bone minerals and the high prevalence of vitamin D deficiency in women are important contributing factors to the higher incidence of hip fractures in women than in men in Iran [39, 40].

We found that age, male sex, diabetes, and cancer increase the risk of mortality in the first year following hip fracture which is consistent with the findings from other studies [41–44]. We also observed a significantly higher risk of re-fracture in those who had been diagnosed with cancer at the time of their first hip fracture which is supported by existing literature [45, 46]. The interviews were conducted a year later, sometimes with patients’ relatives, which could affect the quality of the collected data. Given the retrospective cohort design of our study, we were limited by the available clinical data in the hospital records. A high percentage of about 36% missing data on the “type of treatment approach” variable (see Table 1) shows that hospital medical records may not have been optimally complete. Although we tried to account for this in our regression models (See Table S3 and S4), we could not be sure that our Cox models provided completely unbiased risk estimates for the potential risk factors.

To the best of our knowledge, this is the first study with a large sample size and a relatively long follow-up period that has examined the mortality and re-fracture in patients with hip fractures due to minor trauma in a large orthopaedic centre in Iran. However, as the setting of the study was a large referral orthopaedic hospital in the capital city of Tehran, the generalization of the results to the whole of the country should be done with caution. The results of this study can help us with any evaluation of the impact of healthcare interventions in reducing mortality and re-fracture incidence in the future.

Based on our findings we recommend the implementation of programs for early detection, care, and treatment of people with a history of a hip fracture. Setting up a fracture Liaison Service (FLS) in the hospital could help prevent re-fractures.

### Electronic supplementary material

Below is the link to the electronic supplementary material.

Supplementary Material 1

## Data Availability

All data generated or analysed during this study are included in this published article.
