# Supplementary material for: Mortality and re-fracture rates in low trauma hip fracture
Source: BMC Geriatr. 2024 Apr 30;24:381. doi: 10.1186/s12877-024-04950-1 (PMC11059755; doi:10.1186/s12877-024-04950-1)
Supplement: Supplementary file 1 — Supplementary Material 1 [file 12877_2024_4950_MOESM1_ESM.docx]

Table S1: General characteristics of the cohort of patients with hip fractures caused by low trauma who were referred to Shafa-Yahyaian Hospital between 2013-2019 by the death in the first year

|  | |  | | | | Alive in the first year  N=796 | Died in the first year  N=149 | Total  N =945 | p-value |
| --- | --- | --- | --- | --- | --- | --- | --- | --- | --- |
| Age group | |  | | | |  |  |  | <0.001 |
|  | 50-59 | | | | | 150(1884%) | 8(5.37%) | 158(16.72%) |  |
|  | 60-69 | | | | | 209(26.26%) | 16(10.74%) | 225(23.81%) |  |
|  | 70-79 | | | | | 252(31.66%) | 34(22.82%) | 286(30.26%) |  |
|  | ≥80 | | | | | 185(23.24%) | 91(61.07%) | 276(29.21%) |  |
| Sex | |  | | | |  |  |  | 0.148 |
|  | Female | | | | | 457(57.41%) | 76(51.01%) | 533(56.40%) |  |
|  | Male | | | | | 339(42.59%) | 73(48.99%) | 412(43.60%) |  |
| Education | |  | | | |  |  |  | 0.033 |
|  | No education | | | | | 301(37.81%) | 71(47.65%) | 372(39.37%) |  |
|  | Primary school | | | | | 99(12.44%) | 23(15.44%) | 122(12.91%) |  |
|  | Secondary school | | | | | 65(8.17%) | 14(9.40%) | 79(8.36%) |  |
|  | Diploma | | | | | 56(7.04%) | 8(5.37%) | 64(6.77%) |  |
|  | College | | | | | 31(3.89%) | 1(0.67%) | 32(3.39%) |  |
|  | Missing | | | | | 244(30.65%) | 32(21.48%) | 276(29.21%) |  |
| Fracture type | |  | | | |  |  |  | 0.953 |
|  | Femoral neck | | | | | 361(45.35%) | 67(44.97%) | 428(45.29%) |  |
|  | Intertrochanteric | | | | | 402(50.5%) | 75(50.34%) | 477(50.48%) |  |
|  | Subtrochanteric | | | | | 33(4.15%) | 7(4.70%) | 40(4.23%) |  |
| Osteoporosis treatment | |  | | | |  |  |  | 0.058 |
|  | Received | | | | | 29(3.64%) | 1(0.67%) | 30(3.17%) |  |
|  | Not received | | | | | 767(96.36%) | 148(99.33%) | 915(96.83%) |  |
| Vitamin D supplementation | | | |  | |  |  |  | <0.001 |
|  | Received | | | | | 157(19.72%) | 9(6.04%) | 166(17.57%) |  |
|  | Not received | | | | | 639(80.28%) | 140(93.96%) | 779(82.43%) |  |
| Calcium supplementation | | | | |  |  |  |  | <0.001 |
|  | Received | | | | | 155(19.47%) | 8(5.37%) | 163(17.25%) |  |
|  | Not received | | | | | 641(80.53%) | 141(94.63%) | 782(82.75%) |  |
| Fracture treatment | |  | | | |  |  |  | <0.001 |
|  | Surgical | | | | | 542(65.83%) | 64(42.95%) | 588(62.22%) |  |
|  | Medical | | | | | 10(1.26%) | 4(2.68%) | 14(1.48%) |  |
|  | No information | | | | | 262(32.91%) | 81(54.36%) | 343(36.30%) |  |
| Underlying diseases | | |  | | |  |  |  |  |
|  | Hypertension | | | | | 185(23.24%) | 43(28.86%) | 228(24.13%) | 0.141 |
|  | Stroke | | | | | 31(3.89%) | 10(6.71%) | 41(4.34%) | 0.121 |
|  | Myocardial infarction | | | | | 8(1.01%) | 1(0.67%) | 9(0.95%) | 0.700 |
|  | Kidney disease | | | | | 6(0.75%) | 3(2.01%) | 9(0.95%) | 0.146 |
|  | Thyroid disease | | | | | 30(3.77%) | 9(6.04%) | 39(4.13%) | 0.201 |
|  | Cancer | | | | | 24(3.02%) | 13(8.72%) | 37(3.92%) | 0.001 |
|  | Diabetes | | | | | 108(13.57%) | 26(17.45%) | 134(14.18%) | 0.213 |
| Re-fracture at the first year | | |  | | |  |  |  | <0.001 |
|  | Re-fracture | | | | | 42(6.58%) | 29(100%) | 71(10.64%) |  |
|  | No Re-fracture | | | | | 596(93.42%) | 0(0) | 596(89.36%) |  |

The baseline comparisons show that those who died in the first year were more likely to be older, have lower educational achievements, receive vitamin D and calcium supplementation, have cancer disease, and break their bones again in the first year following their previous fracture.

Table S2: General characteristics of the cohort of patients with hip fractures caused by low trauma who were referred to Shafa-Yahyaian Hospital between 2013-2019 and responded to the follow up call by the occurrence of re-fracture at the first year of the follow-up

|  | |  | | | No Re-fracture during the first year of follow-up  N=638 | Re-fracture during the first year of follow-up  N=29 | Total  N =667 | p-value |
| --- | --- | --- | --- | --- | --- | --- | --- | --- |
| Age group | |  | | |  |  |  | 0.246 |
|  | 50-59 | | | | 108(16.93%) | 2(6.90%) | 110(16.49%) |  |
|  | 60-69 | | | | 153(23.98%) | 7(24.14%) | 160(23.99%) |  |
|  | 70-79 | | | | 188(29.47%) | 13(44.83%) | 201(20.13%) |  |
|  | ≥80 | | | | 189(29.62%) | 7(24.14%) | 196(29.39%) |  |
| Sex | |  | | |  |  |  | 0.157 |
|  | Female | | | | 355(55.64%) | 20(68.97%) | 375(56.22%) |  |
|  | Male | | | | 283(44.36%) | 9(31.03%) | 292(43.78%) |  |
| Education | |  | | |  |  |  | 0.054 |
|  | No education | | | | 354(55.49%) | 18(62.07%) | 372(55.77%) |  |
|  | Primary school | | | | 117(18.34%) | 3(10.34%) | 120(17.99%) |  |
|  | Secondary school | | | | 75(11.76%) | 4(13.79%) | 79(11.84%) |  |
|  | Diploma | | | | 64(10.03%) | 0(0%) | 64(9.60%) |  |
|  | College | | | | 28(4.39%) | 4(13.79%) | 32(4.80%) |  |
| Fracture type | |  | | |  |  |  | - |
|  | Femoral neck | | | | 294(46.08%) | 10(34.48%) | 304(45.58%) |  |
|  | Intertrochanteric | | | | 317(49.69%) | 15(51.72%) | 332(49.78%) |  |
|  | Subtrochanteric | | | | 27(4.23%) | 4(13.79%) | 31(4.65%) |  |
| Osteoporosis treatment | |  | | |  |  |  | 0.459 |
|  | Received | | | | 26(4.08%) | 2(6.90%) | 28(4.20%) |  |
|  | Not received | | | | 612(95.92%) | 27(93.10%) | 639(95.80%) |  |
| Vitamin D supplementation | | |  | |  |  |  | 0.698 |
|  | Received | | | | 152(23.82%) | 6(20.69%) | 158(23.69%) |  |
|  | Not received | | | | 486(76.18%) | 23(79.31%) | 509(76.31%) |  |
| Calcium supplementation | | | |  |  |  |  | 0.445 |
|  | Received | | | | 149(23.35%) | 5(17.24%) | 154(23.09%) |  |
|  | Not received | | | | 489(76.65%) | 24(82.76%) | 513(76.91%) |  |
| Fracture treatment | |  | | |  |  |  | 0.735 |
|  | Surgical | | | | 416(65.20%) | 19(65.52%) | 435(65.22%) |  |
|  | Medical | | | | 13(2.04%) | 0(0%) | 13(1.95%) |  |
|  | No information | | | | 209(32.76%) | 10(34.48%) | 219(32.83%) |  |
| Underlying diseases | |  | | |  |  |  |  |
|  | Hypertension | | | | 207(32.45%) | 8(27.59%) | 215(32.23%) | 0.584 |
|  | Stroke | | | | 40(6.27%) | 1(3.45%) | 41(6.15%) | 0.536 |
|  | Myocardial infarction | | | | 7(1.10%) | 2(6.90%) | 9(1.35%) | 0.008 |
|  | Kidney disease | | | | 7(1.10%) | 1(3.45%) | 8(1.20%) | 0.255 |
|  | Thyroid disease | | | | 36(5.64%) | 2(6.90%) | 38(5.70%) | 0.776 |
|  | Cancer | | | | 29(4.55%) | 4(13.79%) | 33(4.95%) | 0.025 |
|  | Diabetes | | | | 118(18.50%) | 8(27.59%) | 126(18.89%) | 0.221 |
| Death at the first year | |  | | |  |  |  | 0.123 |
|  | Alive | | | | 523(81.97%) | 27(93.10%) | 550(82.46%) |  |
|  | Dead | | | | 115(18.03%) | 2(6.90%) | 117(17.54%) |  |

The baseline comparisons show that those who developed a re-fracture in the first year were more likely to have a history of myocardial infarction and cancer.

Table S3 Hazard ratios and their 95% confidence interval for death in the first year following hip fracture caused by low trauma derived from Cox proportional hazard regression model.

| **Variables in the final model** | **Hazard ratio** | **95% confidence interval** | **P-value** |
| --- | --- | --- | --- |
| Age | 1.09 | 1.07 – 1.11 | **<0.001** |
| Gender (Male: Female) | 1.62 | 1.16 – 2.25 | **0.004** |
| Hypertension | 1.09 | 0.74 – 1.60 | 0.64 |
| History of stroke | 1.31 | 0.65 – 2.64 | 0.44 |
| Chronic kidney failure | 1.22 | 0.36 – 4.14 | 0.745 |
| Thyroid disease | 1.38 | 0.66 – 2.88 | 0.38 |
| Cancer | 3.87 | 2.11 – 7.11 | **<0.001** |
| Diabetes | 1.65 | 1.05 – 2.58 | **0.02** |
| Medical treatment approach vs surgical | 1.78 | 0.64 – 4.95 | 0.26 |

Age, male sex, cancer, and diabetes are independent risk factors for death in the first year in Cox proportional hazard regression modeling.

Table S4 Hazard ratios and their 95% confidence interval for Re-fracture in the first year following hip fracture caused by low trauma derived from Cox proportional hazard regression model.

| **Variables in the final model** | **Hazard ratio** | **95% confidence interval** | **P-value** |
| --- | --- | --- | --- |
| Age | 1.02 | 0.98 – 1.06 | 0.17 |
| Gender (Male: Female) | 0.68 | 0.30 – 1.54 | 0.36 |
| History of myocardial infarction | 3.80 | 0.82 – 17.44 | 0.08 |
| Cancer | 3.52 | 1.16 – 10.71 | **0.02** |
| Diabetes | 1.50 | 0.64 – 3.50 | 0.34 |

Cancer is an independent risk factor for re-fracture in the first year in Cox proportional hazard regression modeling.

Table S5 Survival (fracture-free survival for re-fracture) table in the cohort of study participants.

|  | Year of follow up | Persons at risk at the beginning of the interval | Event | Censored | Survival probability | 95% Confidence Interval |
| --- | --- | --- | --- | --- | --- | --- |
| Deaths during the follow-up |  |  |  |  |  |  |
|  | Year 1 | 945 | 149 | 0 | 0.84 | 0.81 – 0.86 |
|  | Year 2 | 796 | 40 | 162 | 0.79 | 0.76 – 0.81 |
|  | Year 3 | 594 | 36 | 112 | 0.74 | 0.71 – 0.77 |
|  | Year 4 | 446 | 21 | 104 | 0.70 | 0.66 – 0.73 |
|  | Year 5 | 321 | 19 | 111 | 0.65 | 0.61 – 0.68 |
|  | Year 6 | 191 | 11 | 71 | 0.60 | 0.56 – 0.64 |
|  | Year 7 | 109 | 6 | 71 | 0.55 | 0.49 – 0.60 |
| Refracture during the follow-up |  |  |  |  |  |  |
|  | Year 1 | 667 | 29 | 115 | 0.93 | 0.93 - 0.96 |
|  | Year 2 | 523 | 21 | 142 | 0.88 | 0.89 - 0.92 |
|  | Year 3 | 360 | 7 | 109 | 0.85 | 0.87 - 0.91 |
|  | Year 4 | 244 | 4 | 72 | 0.83 | 0.86 - 0.89 |
|  | Year 5 | 168 | 7 | 73 | 0.77 | 0.82 - 0.86 |
|  | Year 6 | 88 | 1 | 45 | 0.75 | 0.81 - 0.85 |
|  | Year 7 | 42 | 1 | 32 | 0.69 | 0.77 - 0.84 |
